# A picorna-like virus associated with *Galba truncatula* (Gastropoda, Lymnaeidae)

**DOI:** 10.1128/mra.00382-26

**Published:** 2026-10-09

**Authors:** Roxane Bories, Laurine Robert-Capraro, Giovanni Begliomini, Thomas Bigot, Dominique Faugere, Pilar Alda, Ignacio G. Bravo, Nolwenn M. Dheilly, Elodie Chapuis

**Affiliations:** 1 MIVEGEC, University of Montpellier, CNRS, IRD 27037, Montpellier, France; 2 Pathogen Discovery Laboratory, Institut Pasteur, Université Paris Cité 555089, Paris, France; 3 Bioinformatics And Biostatistics Hub, Institut Pasteur, Universite Paris Cité 555089, Paris, France; 4 Centro de Recursos Naturales Renovables de la Zona Semiárida (CERZOS), Universidad Nacional del Sur-CONICET 28249, Bahía Blanca, Argentina; Katholieke Universiteit Leuven, Leuven, Belgium

**Keywords:** freshwater snails, picorna-like virus, metatranscriptomics, RNA virus, *Galba truncatula*, genome sequence

## Abstract

Freshwater snails host diverse microorganisms, yet their viruses remain poorly documented. Here, we report the near-complete genome of a picorna-like virus associated with the freshwater snail *Galba truncatula*, recovered using metatranscriptomics. The 9,166-nt genome encodes structural and nonstructural polyproteins and provides a genomic resource for gastropod-associated viruses.

## ANNOUNCEMENT

Freshwater molluscs remain underrepresented in viral databases despite extensive metatranscriptomic surveys of invertebrates. Here, we report a near-complete genome of a picorna-like virus associated with *Galba truncatula* collected in Argentina in 2024.

Snails were collected from six sites (sampling localities): Establecimiento Amelia del Carmen (35.790833°S, 69.667500°W), Acequia Dique (35.537500°S, 69.626389°W), Propiedad Palauco (35.825278°S, 69.499167°W), Acequia 25 de Mayo (34.588611°S, 68.543056°W), Valle Las Leñas (35.197778°S, 70.053611°W), and Cerro Torrecillas (35.109167°S, 70.090278°W). Tissues were preserved in RNAlater and homogenized using a TissueLyzer with two 3 mm stainless-steel beads for 30 s at 30 Hz. Total RNA was extracted using the Monarch Total RNA Miniprep Kit (New England Biolabs), without additional DNase treatment. RNA from five individuals per site was pooled, yielding six pools. Pooled RNA was purified using the RNeasy MinElute Cleanup Kit (Qiagen), and quality was assessed using an Agilent 2100 Bioanalyzer. Libraries were prepared using the SMARTer Stranded Total RNA-Seq Kit v3–Pico Input Mammalian (Takara Bio) and sequenced by the Biomics Platform (Institut Pasteur, Paris) on an Illumina NextSeq 2000 (2 × 100 bp paired-end), yielding 41.0–72.3 million paired reads per sample.

Reads were processed using Microseek (1), including quality trimming, normalization, *de novo* assembly, and open reading frame (ORF) prediction, with viral RNA detected by similarity searches against RVDB-prot (2). Genome assembly was validated by read mapping using CLC Genomics Workbench (Qiagen).

**Fig 1:**
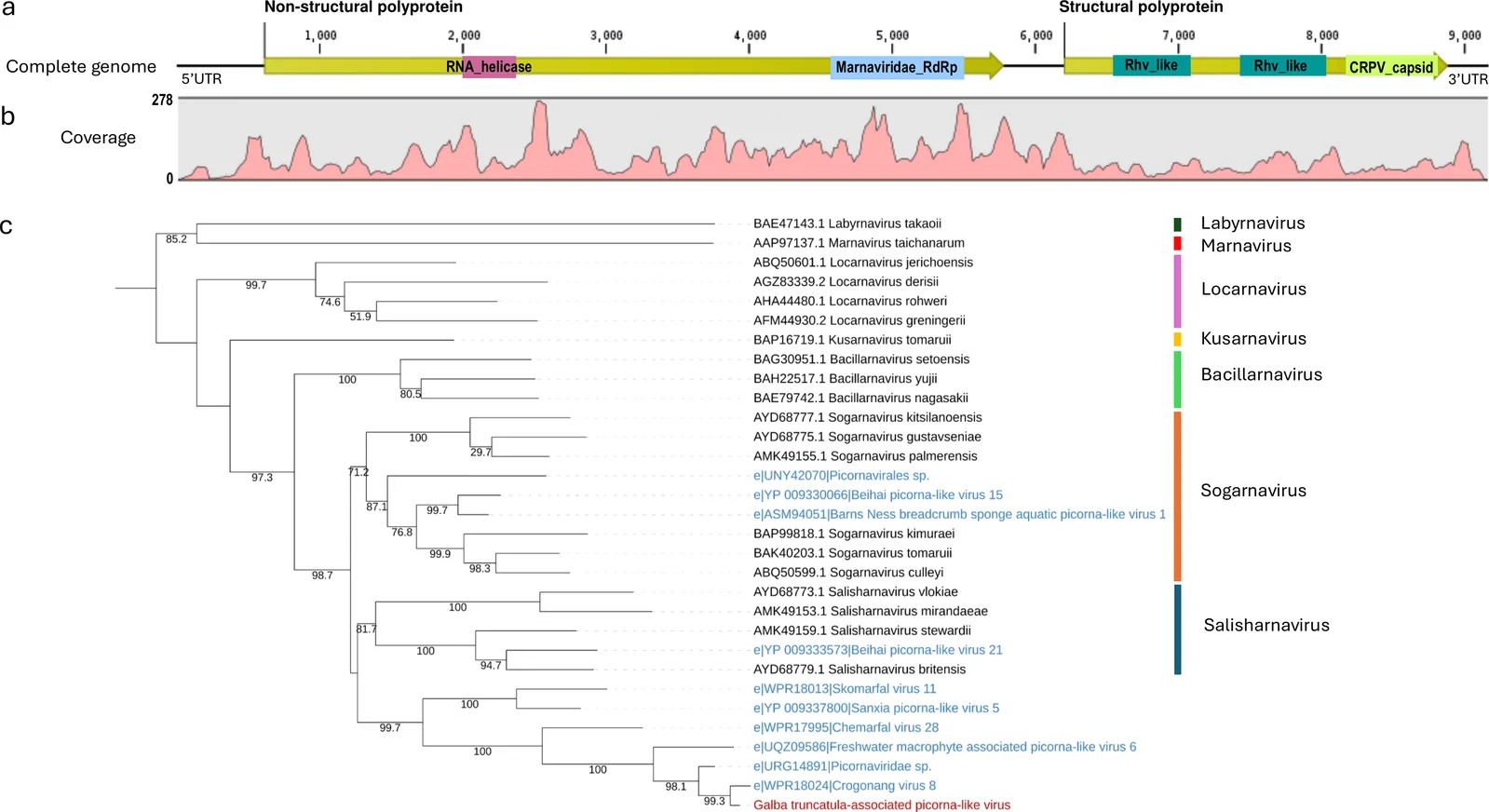
Genome structure, sequencing coverage, and phylogenetic relationships of *Galba truncatula*–associated picorna-like virus. (**a**) Genome organization of the near-complete genome. (**b**) Coverage of total RNA reads that mapped to the genome. (**c**) Maximum-likelihood phylogenetic tree inferred from the RNA-dependent RNA polymerase (RdRp) domain of representative viral species from the family *Marnaviridae*. Sequences of ICTV-recognized viruses and the 10 best BLAST hits retrieved from RVDB-prot (4) were included in the analysis (blue). ICTV-recognized genera are provided next to the tree. Amino acid sequences were aligned using MAFFT v7.525 (5). The phylogenetic tree was reconstructed using IQ-TREE v2.4.0 (6) with the SH-aLRT branch test (7) and 10,000 replicates (bootstrap support indicated alongside the branches). ModelFinder identified Q.pfam + F + I + R4 as the best-fitting amino acid substitution model according to the Bayesian Information Criterion (8). The near-complete genome identified in this study is indicated in red and bold (accession number PX912124).

A near-complete picorna-like viral genome was assembled from one pool (site 3), with an average coverage of 85× (Fig. 1a and b). The 9,166-nt genome shared 78% nucleotide identity and 88% sequence overlap with Crogonang virus 8 isolate CGH03 (OR270229), identified in freshwater mussels in Oregon, USA (3). Its 705-nt 5′UTR and 111-nt 3′UTR were longer than the corresponding 78- and 53-nt UTRs of CGH03, while predicted ORFs had the same lengths.

ORFs were predicted using ORFfinder and annotated by BLASTp against the National Center for Biotechnology Information RefSeq viral proteins and InterProScan. The genome contained 2 ORFs encoding a nonstructural (4,617 nt; 1,538 aa) and a structural (2,685 nt; 894 aa) polyprotein. The nonstructural polyprotein contains RNA helicase (pfam00910) and *Marnaviridae* RNA-dependent RNA polymerase (RdRp; cd23195) domains and shares 90.6% amino acid identity with the CGH03 homolog. The structural polyprotein contains three Jellyroll-fold domains, including two rhinovirus-like picornavirus capsid domains (cd00205) and a Cricket Paralysis virus-like capsid domain (pfam08762), and shared 81% identity with the CGH03 homolog (Table 1).

**TABLE 1:** Pairwise nucleotide and amino acid sequence identities between *Galba truncatula*–associated picorna-like virus (PX912124) and related viruses calculated using Clustal Omega v1.2.4

Virus	Accession	Host	Geographic origin	Genome length (nt)	Nucleotide identity (%)	Nonstructural polyprotein identity (%)	Structural polyprotein identity (%)
*Galba truncatula*-associated picorna-like virus	PX912124	*Galba truncatula*	Argentina	9,166	Reference	Reference	Reference
Crogonang virus eight isolate CGH03	OR270229	*Gonidea angulata*	Oregon, USA	8,478	78.73	90.64	81.32
*Picornaviridae* sp. isolate 374R	ON162285	Reservoir sediment	Heilongjiang, China	>1,072	75.65	84.87	NA^*a*^
Freshwater macrophyte associated picorna-like virus 6	ON125131	Macrophytes collected in freshwater springs	Florida, USA	9,854	57.00	51.93	54.49
Chemarfal virus 28	OR270214	*Margaritifera falcata*	Washington, USA	9,127	52.52	40.30	45.57
Sanxia picorna-like virus 5	NC_033262	Freshwater atyid shrimp	China	8,673	52.73	28.19	38.30
Skomarfal virus 11	OR270223	*Margaritifera falcata*	Washington, USA	8,798	45.86	30.68	34.84

^*a*^ NA, not available.

The RdRp domain, identified with hmmscan, was compared with the International Committee for the Taxonomy of Viruses-recognized *Marnaviridae* members and the 10 best unclassified-virus hits from RVDB-prot (4) using DIAMOND BLASTp (5), with IQ-TREE (6). Phylogenetic analysis placed the virus within *Marnaviridae* (order *Picornavirales*), in an unclassified clade of viruses (Fig. 1c, Table 1). Based on ICTV species-demarcation thresholds (90% and 75% amino acid identity for RdRp and capsid proteins, respectively), it likely belongs to the same species as Crogonang virus 8. However, their occurrence in distinct hosts (snails and mussels) and locations (Argentina and USA) indicates a broader host and geographic distribution and may support taxonomic separation at the species level.

## Data Availability

The near-complete genome is available in GenBank (PX912124) and raw reads in Sequence Read Archive (SRR38391848) and BioProject (PRJNA1462349).
